# Supplementary material for: Cellular geometry scaling ensures robust division site positioning
Source: Nat Commun. 2019 Jan 21;10:268. doi: 10.1038/s41467-018-08218-2 (PMC6341079; doi:10.1038/s41467-018-08218-2)
Supplement: Supplementary file 1 — Supplementary Information [file 41467_2018_8218_MOESM1_ESM.pdf]

Supplementary information

Cellular geometry scaling ensures robust division site positioning

Ying Gu and Snezhana Oliferenko

This file includes:

Supplementary figures 1 to 5
Supplementary tables 1 and 2

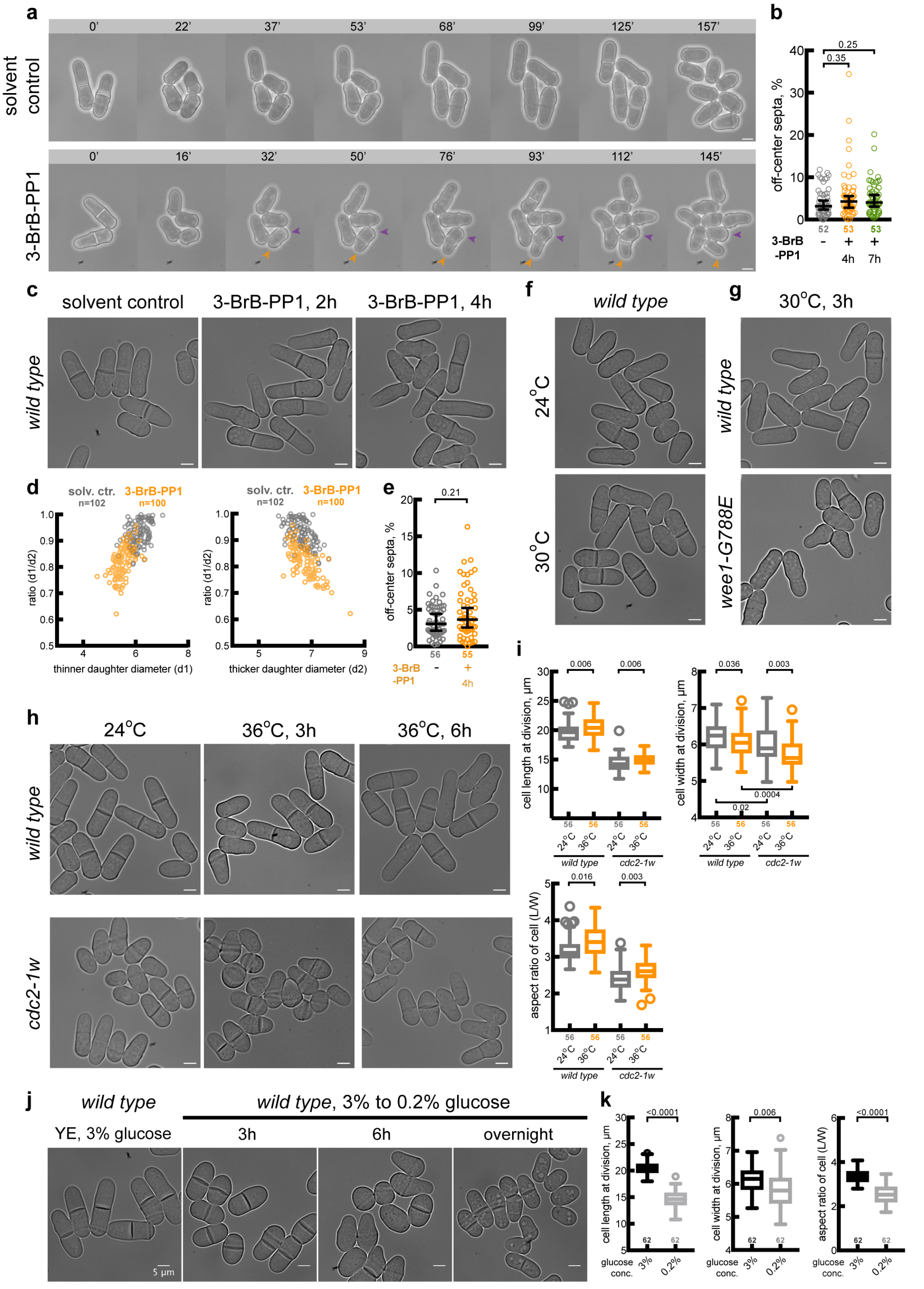

Supplementary figure 1. *S. japonicus* scales its geometry in a variety of experimental conditions. (**a**) Time-lapse montages of maximum intensity z-projected bright-field images of *S. japonicus wee1-as8* cells in methanol

(solvent control) (*top*) or 20 μM 3-BrB-PP1 (*bottom*). Methanol or 3-BrB-PP1 was administered one hour prior to imaging. Orange arrowheads point toward a hyperpolarizing cell end whereas magenta arrowheads indicate the other, thicker cell end. (**b**) A plot summarizing the accuracy of division plane positioning in methanol- or 3-BrB-PP1-treated *wee1-as8* cells shown in Fig. 1a. (**c**) *S. japonicus* wild type cells incubated with 20 μM 3-BrB-PP1 for indicated time. (**d**) A 2-hour treatment with 3-BrB-PP1 causes mild hyperpolarization at one cell tip in wild type cells, indicative of off-target effects. Shown are the scatter plots, where either 'thinner' (*top*) or 'thicker' (*bottom*) daughter cell diameter measurements are on x-axis, and the ratios between diameters of the daughters are on y-axis. (**e**) A plot summarizing the accuracy of division plane positioning shown in (**c**). (**f**) Representative images of wild type cells cultured at 24°C (*top*) and 30°C (*bottom*). (**g**) *S. japonicus* wild type and *wee1-G788E* mutant (*bottom*) cells shifted from 24°C to 30°C for 3 hours. (**h**) *S. japonicus* wild type (*top*) and *cdc2-1w* mutant (*bottom*) cells cultured at 24°C, and upon the shift to 36°C for 3 and 6 hours. (**i**) Measurements of cellular length, width and aspect ratio of the wild type as compared to *cdc2-1w* cells at the 6-hour time point, shown in (**h**). (**j**) *S. japonicus* cells grown in YE with 3% glucose and shifted to YE-0.2% glucose for 3, 6 hours and overnight. (**k**) Quantifications of cell length, width and aspect ratio at division in YE with 3% glucose and 0.2% glucose overnight, for cells shown in (**j**). (**c, f**, **g**, **h**, **j**) Shown are single z-plane bright-field micrographs of live cells; scale bars represent 5 μm. (**b** and **e)** Shown as 1D-scatter plots. Deviation from the geometric center of the cell is indicated on y-axis. Black bars represent sample median with error bars indicating 95%

confidence intervals. (**i**, **k**) Quantifications presented as box plots with whiskers calculated by the Tukey method. n indicated in figures; *p* values derived from Kolmogorov-Smirnov test.

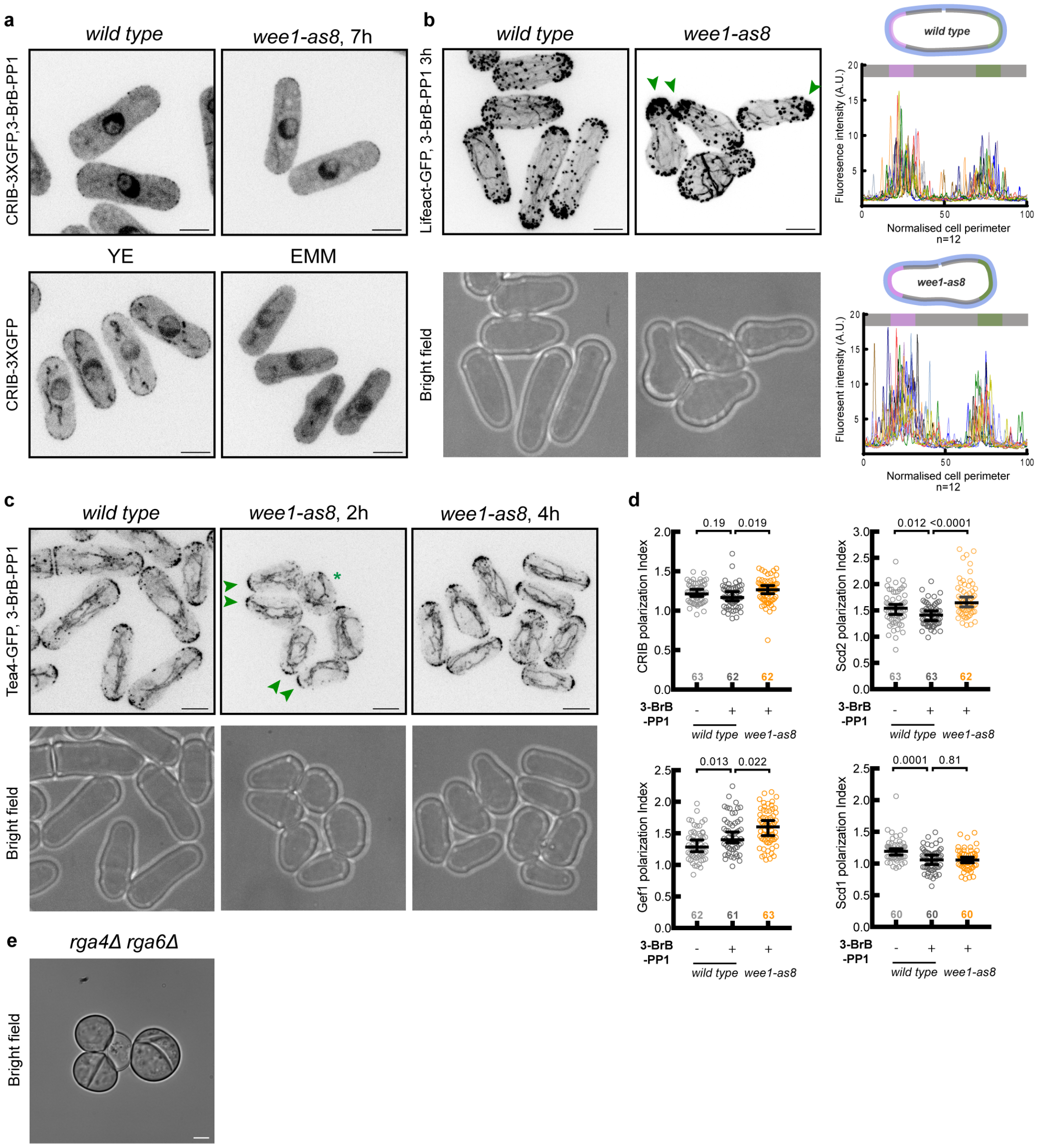

Supplementary figure 2. Localization of cellular polarity factors in control *S. japonicus* cells and during the process of scaling. (**a**) Following scaling, cells do not show relative enrichment of Cdc42 activity at cell tips. Spinning disk confocal micrographs of *S. japonicus* CRIB-3xGFP-expressing wild type and *wee1-as8* cells grown in indicated experimental conditions. (**b**) *S. japonicus* wild type and *wee1-as8* cells expressing LifeAct-GFP and the corresponding bright-field images. Green arrowheads indicate hyperpolarized cell tips. Cortical LifeAct-GFP fluorescence intensities (y-axis) normalized to cell

perimeter (x-axis) (*right*) indicate F-actin enrichment at hyperpolarizing tips in Wee1-inhibited cells. (**c**) 3-BrB-PP1-treated *S. japonicus* wild type and *wee1-as8* cells expressing Tea4-GFP and the corresponding bright-field images. Tea4 is preferentially enriched at the hyperpolarized cell tip in Wee1-inhibited cells (indicated by green arrowheads). Note that Tea4 does not exhibit specific cortical localization in a depolarized Wee1-inhibited cell at a 2-hour time-point (indicated by a green star). (**d**) Graphs showing relative fluorescence intensities of indicated marker proteins at thinner cell tips, for cells shown in Fig. 2c. Shown as 1D-scatter plots. Black bars represent sample median with error bars indicating 95% confidence intervals. n indicated in figures; *p* values derived from Kolmogorov-Smirnov test. (**e**) Single z-plane bright-field micrographs of *S. japonicus rga4*Δ*rga6*Δ cells. Simultaneous removal of both Cdc42 GAPs Rga4 and Rga6 in *S. japonicus* gives rise to virtually spherical cells, indicating cooperation between these two GAPs in maintaining polarized growth. (**a**-**c**) shown are maximum intensity z-projections of spinning-disk confocal micrographs of cells. (**a**-**c**, **e**) Scale bars represent 5 μm.

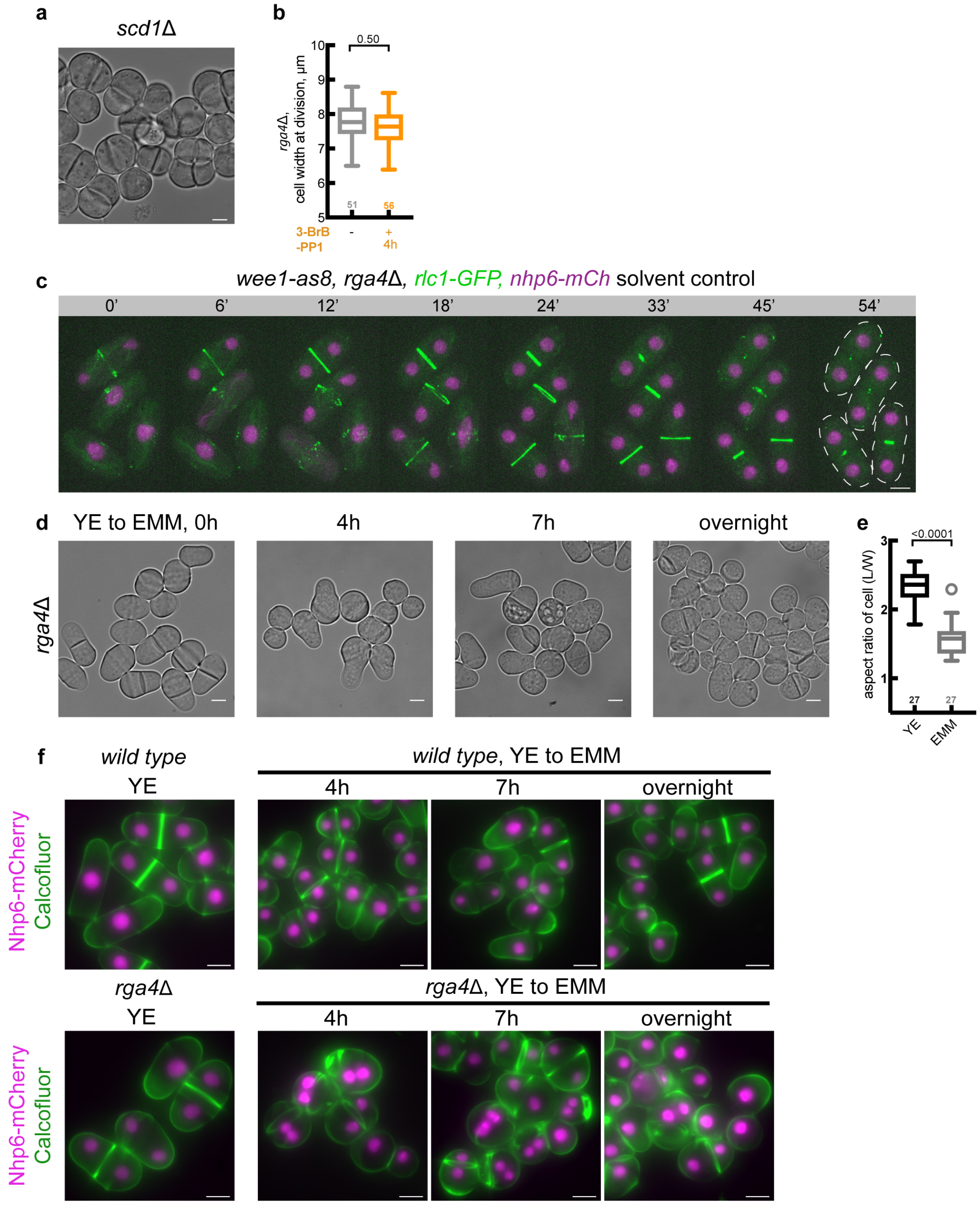

Supplementary figure 3. Rga4 is required for geometrical scaling in *S. japonicus*. (**a**) Representative image of *S. japonicus scd1Δ* cells. *S. japonicus* cells grown on YES agar lacking Cdc42 GEF Scd1 are defective in establishing polarized growth and cannot proliferate in the liquid YES medium. (**b**) Graph representing cell width at division for *rga4Δ S. japonicus* cells treated with solvent or 3-BrB-PP1 for 4 hours. (**c**) Time-lapse montage of

maximum intensity z-projected spinning-disk confocal micrographs of methanol-treated (as solvent control) *wee1-as8 rga4Δ S. japonicus* cells expressing Rlc1-GFP and Nhp6-mCherry. Myosin complexes assemble into medially positioned rings in these cells. Cell boundaries are outlined by white dashed lines. (**d**) *S. japonicus rga4Δ* cells after the shift from YE to EMM medium for 0, 4 and 7 hours followed by overnight incubation in EMM. (**e**) Graph showing cellular aspect ratio of cells with division septa shown in (**d**) before and after overnight incubation in EMM. (**f**) Pseudocolored maximum intensity z-projected epifluorescence images of wild type (*top*) and *rga4Δ S. japonicus* cells (*bottom*) expressing nuclear Nhp6-mCherry (magenta) stained with Calcofluor White (green) to visualize division septa under indicated conditions. Note that *rga4Δ* cells fail to rescale cell geometry after nutritional shift and display off-center division septa giving rise to bi-nucleated daughter cells after incubation in EMM. (**a** and **d**) Shown are single z-plane bright-field micrographs. (**a**, **c**, **d** and **f**) Scale bars represent 5 μm. (**b**, **e**) Box plots with whiskers; n indicated in figures; *p* values derived from Kolmogorov-Smirnov test.

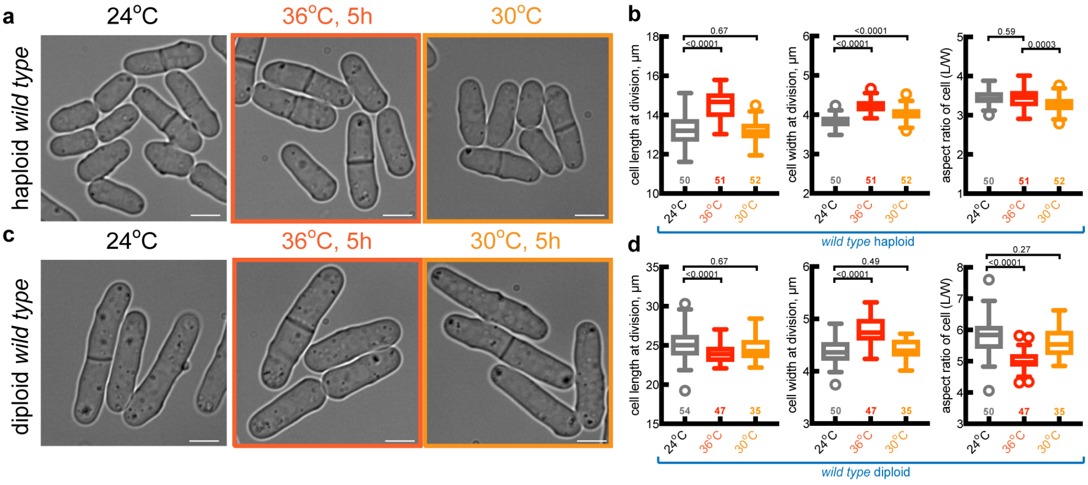

Supplementary figure 4. Analyses of wild type *S. pombe* cells grown at different physiological temperatures. (**a**) *S. pombe* wild type haploid cells grown at 24°C overnight (grey), shifted to 36°C for 5 hours (red) or grown at 30°C overnight (orange). (**b**) Quantifications of cell length, width and aspect ratio at division of wild type *S. pombe* haploid cells at indicated temperatures shown in (**a**). (**c**) *S. pombe* wild type diploid cells grown at 24°C overnight (grey), shifted to 36°C for 5 hours (red) or grown at 30°C for 5 hours (orange). (**d**) Quantifications of cell length, width and aspect ratio at division of wild type *S. pombe* diploid cells at indicated temperatures shown in (**c**). (**a** and **c**) Scale bars represent 5 µm. (**b** and **d**) Presented are box plots with whiskers calculated by the Tukey method. n indicated in figures; *p* values derived from Kolmogorov-Smirnov test.

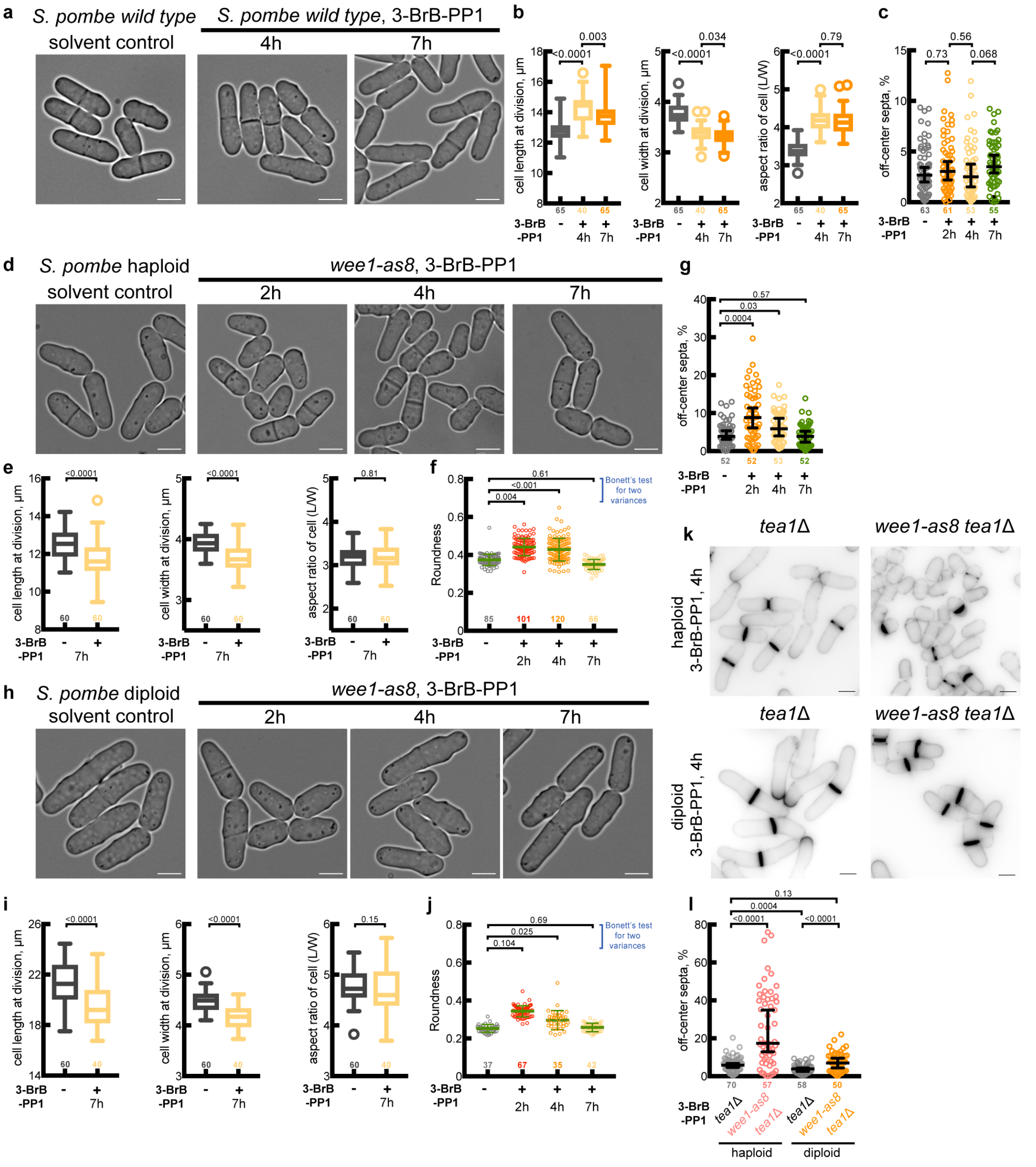

Supplementary figure 5. Analyses of wild type *and wee1-as8 S. pombe* cells grown in the presence of the ATP analog 3-BrB-PP1.(**a**) *S. pombe* wild type cells incubated with 20 µM 3-BrB-PP1 for indicated time. (**b**) Quantifications of cell length, width and aspect ratio at division of cell population represented in (**a**). (**c**) A plot summarizing the accuracy of division plane positioning shown in (**a**). (**d**) *S. pombe wee1-as8* haploid cells incubated with methanol (solvent control) or 20 µM ATP analog 3-BrB-PP1 for 2, 4 and 7 hours. (**e**)

Quantifications of cell length, width and aspect ratio at division after Wee1 inhibition in *S. pombe wee1-as8* haploids shown in (**d**). (**f**) Cell shape at division was assessed using roundness parameter as a proxy, where spherical cells are assigned a roundness value of 1. Shown is a plot of the distribution of cell roundness values within an *S. pombe wee1-as8* haploid population treated with 20 μM 3-BrB-PP1 for indicated time points shown in (**d**). (**g**) A plot summarizing the accuracy of division plane positioning in methanol- or 20 μM 3-BrB-PP1-treated *S. pombe wee1-as8* haploid cells at different time points shown in (**d**). Deviation from the geometric center of the cell is indicated on y-axis. (**h**) *S. pombe wee1-as8* diploid cells incubated with methanol or 20μM 3-BrB-PP1 for 2, 4 and 7 hours. (**i**) Quantifications of cell length, width and aspect ratio at division after Wee1 inhibition in *S. pombe wee1-as8* diploid cells shown in (**h**). (**j**) A plot showing the distribution of cell roundness values within an *S. pombe wee1-as8* diploid population treated with 20 μM 3-BrB-PP1 for indicated time points shown in (**h**). (**k**) Maximum intensity z-projections of epifluorescence images of Calcofluor White-stained live *S. pombe tea1Δ* and *wee1-as8 tea1Δ* haploid (t*op*) and diploid (*bottom*) cells incubated with 20 μM 3-BrB-PP1 for 4 hours. (**l**) A plot summarizing the accuracy of division plane positioning in 3-BrB-PP1-treated cells shown in (**k**). (**a**, **d**, **h**) Shown are single z-plane bright-field micrographs. Scale bars represent 5 μm. (**b**, **e**, **i**) Presented are box plots with whiskers; n indicated in figures; *p* values derived using Kolmogorov-Smirnov test. (**f**, **j**) *p* values derived using Bonett's test for two variances. Green bars represent sample mean. Error bars represent standard deviation. (**c**, **g**, **l**) Shown are 1D scatter plots;_black bars represent sample median with error bars indicating 95%

confidence intervals. n indicated in figures; p values derived using Kolmogorov-Smirnov test.

Schizosaccharomyces japonicus	
**Collection No.**	**Genotype**
SOJ5	NIG2028 matsj-P2028 h- (prototroph)
SOJ2876	*wee1-as8*::kanR h- (prototroph)
SOJ3414	*wee1-G788E*::*ura4+ ura4sj-D3* h-
SOJ3312	*cdc2-1w*::*ura4+ ura4sj-D3*
SOJ1345	CRIB-3xGFP::*ura4+ ura4sj-D3* h+
SOJ2443	*wee1-as8::ura4+* CRIB-3xGFP::*ura4+ ura4sj-D3* h-
SOJ638	Lifeact-GFP::*ura4+ nhp6-mCherry::ura4 ura4sj-D3*
SOJ2459	*wee1-as8::ura4+* Lifeact-GFP::*ura4+ nhp6-mCherry::ura4 ura4sj-D3* h-
SOJ2465	*tea4-GFP::ura4+ nhp6-mCherry::ura4+ ura4sj-D3*
SOJ2464	*wee1-as8::ura4+ tea4-GFP::ura4+ ura4sj-D3*
SOJ2504	*gef1-mNeonGreen::kanR nhp6-mCherry:ura4+ ura4sj-D3*
SOJ2503	*wee1-as8::ura4+ gef1-mNeonGreen::kanR ura4sj-D3*
SOJ2507	*scd1-mNeonGreen::kanR nhp6-mCherry:ura4+ ura4sj-D3*
SOJ2506	*wee1-as8::ura4+ scd1-mNeonGreen::kanR ura4sj-D3*
SOJ2509	*rga4-mNeonGreen::kanR nhp6-mCherry:ura4+ ura4sj-D3* h+
SOJ2510	*wee1-as8::ura4+ rga4-mNeonGreen::kanR ura4sj-D3* h+
SOJ2602	*rga6-GFP::ura4+ ura4sj-D3* h+
SOJ2622	*wee1-as8::ura4+ rga6-GFP::ura4+ rlc1-mCherry::ura4+*::kanR *ura4sj-D3* h+
SOJ2825	*scd2-mNeonGreen::ura4+ ura4sj-D3* h-
SOJ2834	*wee1-as8::*kanR *scd2-mNeonGreen::ura4+ ura4sj-D3* h-
SOJ3615	*gef1Δ::ura4+ ura4sj-D3*
SOJ3316	*wee1-as8::*kanR *gef1Δ::ura4+ ura4sj-D3*
SOJ3269	*rga4Δ*::kanR (prototroph)
SOJ2477	*wee1-as8::ura4+ rga4Δ::*kanR *ura4sj-D3*
SOJ3608	*rga6Δ*::natR (prototroph)
SOJ3317	*wee1-as8::ura4+ rga6Δ::*natR *ura4sj-D3*
SOJ3628	*rga4Δ:*kanR *gef1Δ:ura4+ ura4sj-D3*
SOJ3336	*wee1-as8::ura4+ rga4Δ::*kanR *gef1Δ::ura4+ ura4sj-D3*
SOJ1360	*rga4Δ::ura4+ pom1-GFP::ura4+*::kanR *ura4sj-D3*
SOJ2532	*wee1-as8::ura4+ rga4Δ::ura4+ pom1-GFP::ura4+*::kanR *ura4sj-D3*
SOJ3324	*wee1-as8::ura4+ rga4Δ::*kanR *rlc1-GFP::*kanR *nhp6-mCherry::ura4+ ura4sj-D3*
SOJ3329	*wee1-as8::*kanR *scd1Δ::ura4+ ura4sj-D3*
SOJ3338	*rga4Δ::*kanR *rga6Δ::*natR (prototroph)
SOJ3610	*nhp6-mCherry::ura4+ ura4sj-D3*
SOJ3607	*rga4Δ:*kanR *nhp6-mCherry:ura4+ ura4sj-D3*
SOJ3420	*wee1-G788E::*kanR *rga4Δ:ura4+ ura4sj-D3*
SOJ3686	*wee1-G788E::*kanR *rga4Δ:*kanR *nhp6-mCherry:ura4+ ura4sj-D3*
Schizosaccharomyces pombe	
**Collection No.**	**Genotype**
SO7812	wild type strain 972 h-
From Kaz Shiozaki	CRIB-3xGFP::*ura4+ ura4-294 leu1-32* h-
From Fulvia Verde	*rga4-GFP::*kanR *ade6-704 leu1-32 ura4D-18* h+
From Mohan Balasubramanian	*wee1-50 leu1-32* h-
SO8327	*wee1-50 ade6-210/216 ura4-D18? leu1-32?* (diploid)
SO7404	*tea1Δ::ura4+ ade6-21x leu? ura?* h+

SO7405	*tea1Δ::ura4+ ade6-21x leu? ura?* h-
From Iain Hagan	*wee1-as8 (V644G, M700F)*::kanR h-
SO8235	*wee1-as8*::kanR *ade6-210/216 leu1-32?* (diploid)
SO8286	*wee1-as8*::kanR *tea1Δ::ura4+ ade6-210 ura4-D18 leu1-32* h-
SO8298	*wee1-as8*::kanR *tea1 Δ ::ura4+ ade6-210/216 ura4-D18? leu1-32?* (diploid)
SO8243	*ade6-210/216 ura4-D18 leu1-32* (diploid)
SO8308	*wee1-50 tea1 Δ ::ura4+ ade6-216 ura4-D18 leu1-32* h-
SO8309	*wee1-50 tea1 Δ ::ura4+ ade6-210/216 ura4-D18 leu1-32?* (diploid)

Supplementary table 1.

List of *Schizosaccharomyces japonicus* and *Schizosaccharomyces pombe* strains

ATACTCGAGGGCATGGTTTGGCTGAG	XhoI_promoter_pak1_sj (Schizosaccharomyces japonicus) fwd
TATCCCGGGCTTGAAGACTAAAAGCCTG	SmaI_promoter_pak1_sj rev
ATACCCGGGATGACTAGTGCAAGTATTAC	SmaI_gic2_sc (Saccharomyces cerevisiae) fwd
TACCCGGG**cc**CTTATTTTCGTGCGATCTTG	SmaI_gic2_sc rev
TATCCCGGGCAGCTTGTTAATTAAAC	SmaI_gef1_sj C-terminus tagging fwd
ATAGGATCCGGCCTCCTTCAGCAGAT	BamHI_gef1_sj C-terminus tagging rev
TATGGGCCCGAGGCCTAACTTTATGATG	ApaI_gef1_sj 3'UTR fwd
ATACCCGGGACGACACGGTTTCA	SmaI_gef1_sj 3'UTR rev
TATCCCGGGAATTATTCAAAGAACCT	SmaI_rga4_sj C-terminus tagging fwd
ATAGGATCCGAAAAGCTCCTCAACGTG	BamHI_rga4_sj C-terminus tagging rev
TATGGGCCCAATCATAAGAAACATATG	ApaI_rga4_sj 3'UTR fwd
ATACCCGGGAGGAGTGACAGAGT	SmaI_rga4_sj 3'UTR rev
TATCCCGGGAAAATGAGCAAGACTG	SmaI_scd1_sj C-terminus tagging fwd
ATAGGATCCTGGGGCTAAGCTTGTGAA	BamHI_scd1_sj C-terminus tagging rev
TATGGGCCCATAATGCTTGTTCTAC	ApaI_scd1_sj 3'UTR fwd
ATACCCGGGAGTTGAATCAGAATC	SmaI_scd1_sj 3'UTR rev
TATCTCGAGAAAGTTCGGCTTG	XhoI_rga6_sj C-terminus tagging fwd
ATACCCGGGAGTTTTTTTCTTCTTAAAAAGTC	SmaI_rga6_sj C-terminus tagging rev
TATGGGCCCTACCGACTTTTTGAC	ApaI_rga6_sj 3'UTR fwd
ATACTCGAGGTTCAAGGTAATTCC	XhoI_rga6_sj 3'UTR rev
TATATCGATTCGCGTATATCCCTT	ClaI_scd2_sj C-terminus tagging fwd
ATAGGATCCTTCCGAAATGAAGCCGTC	BamHI_scd2_sj C-terminus tagging fwd
TATGGGCCCGGTTTGCTTGTTTCATG	ApaI_scd2_sj 3'UTR fwd
ATAATCGATAGGGAATAATACGTTGTCG	ClaI_scd2_sj 3'UTR rev
ATACTCGAGTTATCACGTACGTTTTC	XhoI_tea4_sj C-terminus tagging fwd
AAAAAGATCTTAAAGATTTCACAGCAGTT	BglII_ tea4_sj C-terminus tagging fwd
TATGGTACCTAAAAACAATCGGTTCT	KpnI_ tea4_sj 3'UTR fwd
ATACTCGAGATGTGCATTTGTCCAAA	XhoI_ tea4_ sj 3'UTR rev
TATGGGCCCGTGAAAAAACTAAAAG	ApaI_wee1-G788E_sj mutagenic fragment 1 fwd
CTTCGTAAATTGTTGC**tt**CGGCCTTGCGTC	wee1-G788E_sj mutagenic fragment 1 rev
**aa**GCAACAATTTACGAAGGA	wee1-G788E_sj_mutagenic fragment 2 fwd
AAACCCGGGACATTAGACATATCAAGG	SmaI_wee1_sj_3'UTR rev
TATGGGCCCACTCCCATTCATGCCTCT	ApaI_wee1-as8_sj fragment 1 fwd
ACCTTGCATGTACAAGTAG	wee1-as8_sj fragment 1 rev
TGGCTACTTGTACATGCAA**ggt**GAGCTTTGCGAAAAT GGAAG	wee1_as8_sj mutagenic fragment fwd
GGTGCCCTCAAACGTAAT**gaa**AATGTTTGCGGGCTT CAAATC	wee1_as8_sj mutagenic fragment fwd
TTCATTACGTTTGAGGGCA	wee1-as8_sj fragment 2 fwd
ATACCCGGGTCAGAAGTAGGAAGCCAG	SmaI_wee1-as8_sj fragment 2 rev
TATGGTACCTGGCTTCCTACTTCTGA	KpnI_wee1_as8_sj 3'UTR fwd
ATAGGGCCCGTTTCTCGTAACAGCTAT	ApaI_wee1_as8_sj '3UTR rev
TATCCCGGGTATTGAGAGTCGGT	SmaI_cdc2_sj 5'UTR to amplify ORF fwd
ATACCGCGGCAAGTGTGACAGGAGTG	SacII_cdc2_sj 3'UTR to amplify ORF rev
TATGGGCCCACCCTCTTTCCTTGTTC	ApaI_cdc2_sj 3'UTR fwd

ATACCCGGGAACATTTTCTGCACCACCA	SmaI_cdc2_sj 3'UTR rev
GCGAGTTTCAGATTGtCTTCTTTGTCGATCAGCAA	cdc2_1w_sj mutagenic fragment 1 rev
AAGaCAATCTGAAACTCGCCGA	cdc2_1w_sj mutagenic fragment 2 fwd
TATCCCGGGATGCAGCTGCTTC	SmaI_scd1_sj 5'UTR to knockout ORF fwd
ATAGGATCCGTCATCAACGTTGCGAGA	BamHI_scd1_sj 5'UTR to knockout ORF rev
TATGGGCCCATAATGCTTGTTCTAC	ApaI_scd1_sj 3'UTR to knockout ORF fwd
ATACCCGGGAGTTGAATCAGAATC	SmaI_scd1_sj 3'UTR to knockout ORF rev
TATCCCGGGTCTTTACAATTCTCTG	SmaI_gef1_sj 5'UTR to knockout ORF fwd
ATAGGATCCGTGTCGTAAACGTTTGAA	BamHI_gef1_sj 5'UTR to knockout ORF rev
TATGGGCCCGAGGCCTAACTTTATGATG	ApaI_gef1_sj 3'UTR to knockout ORF fwd
ATACCCGGGACGACACGGTTTCA	SmaI_gef1_sj 3'UTR to knockout ORF rev
ATACCCGGGCCGTCAGATCATTG	SmaI_rga4_sj 5'UTR to knockout ORF fwd
ATAGGGCCCGCTTATGAAATGAACGGTG	ApaI_rga4_sj 5'UTR to knockout ORF rev
TATTCTAGATTGACCACGTTGAGGAGC	XbaI_rga4_sj 3'UTR to knockout ORF fwd
ATACCCGGGAGGAGTGACAGAGTTC	SmaI_rga4_sj 3'UTR to knockout ORF rev
TGTTCTAAATCCGTCATATTGGCTATAAAAACTACGT TACGTATACGAAATCTTAATGGACGATACTTCTCTTG CCTCGTgcgccacttctaaataagc	rga4_sj kanMX6 cassette knockout fwd
TCGAACGTCAATCATACCTATCTAGAGTCATAGTAAG GAAACATAACATCATATGTTTCTTATGATTGGGGTTA AGATTAaattcgagctcgtttaaac	rga4_sj kanMX6 cassette knockout rev
CGGTACAGGTTGTGACGTCTTCCAGCACTCACAGGT TTACTTATTTTTTCCATCTTTATCCTGTCATTTTTTGTT CTTCTTGgtttagcttgcctcgtc	rga6_sj natMX6 cassette knockout fwd
AATTATACACTTTCCTTTGCAACACAATATACAGAAA AAGGATTGTCAAAAAGTCGGTAGGGAGGTAATTTAC CACATCATTgatggcggcgttagtat	rga6_sj natMX6 cassette knockout rev
GTTCTCAGCAACTCCTT	rga4_sj 5'UTR genotyping fwd
CACAAGTTATGCATCTGA	rga4_sj ORF genotyping rev
CTACACTCATCCGAGATC	rga4_sj ORF genotyping fwd
TGGAGCACCTCTAGATAT	rga4_sj 3'UTR genotyping rev
GCTTTTATACAACACTGGAAT	rga6_sj 5'UTR genotyping fwd
TCGTTACCTGAGGACGAA	rga6_sj ORF genotyping rev
TCGCCCTTATCGCCTTC	rga6_sj ORF genotyping fwd
AGACGGATGCTTGTAATCA	rga6_sj 3'UTR genotyping rev
TTCACTGCGTCAGCTTGT	gef1_sj 5'UTR genotyping fwd
TCATCGTGAACTGCTCGT	gef1_sj ORF genotyping rev
CAGATGCAGATGTAACTGT	gef1_sj ORF genotyping fwd
GAGTCTCACCTAAACGAAG	gef1_sj 3'UTR genotyping rev
TACTAATCAGCAGCGCTG	scd1_sj 5'UTR genotyping fwd
CACGCGGATGTTTTACAAC	scd1_sj ORF genotyping rev
GCCTTTGAGTATTGCGTGT	scd1_sj ORF genotyping fwd
CTCAGATGAAGTTGTTTCTC	scd1_sj 3'UTR genotyping rev

Supplementary table 2.

List of primers
